# Supplementary material for: The Sec1/Munc18 (SM) protein Vps45 is involved in iron uptake, mitochondrial function and virulence in the pathogenic fungus Cryptococcus neoformans
Source: PLoS Pathog. 2018 Aug 2;14(8):e1007220. doi: 10.1371/journal.ppat.1007220 (PMC6091972; doi:10.1371/journal.ppat.1007220)
Supplement: S1 Table — (PDF) [file ppat.1007220.s001.pdf]

SUPPLEMENTAL INFORMATION

S1 Table: Primer used in this study

Primer name	Sequence
VPS45KO1	GCCCGCAAAGAATGTCATGTC
VPS45KO2	GTTTCTACATCTCTTCCGTGTTAATACAGATATGCCCTGTGGGTTGAACG
VPS45KO3	CATGCTTATGTGAGTCCTCCCCTTCCCGTGCAAGCGGAGTGAGTCTAAG
VPS45KO4	AAGGGCGATGAGATCAAGG
VPS45KO5	GACATCTGTGCCCATGAC
VPS45KO6	CGTTCTCCAGCGAGTAAG
Cassette F	TCTGTATTAACACGGAAGAGATGTAGAAAC
Cassette R	CACGGGAAGGGGAGGACTCACATAAGCATG
CRZ1 KO1	CCCGATTTTGCTTTTTAGTGCGTGG
CRZ1 KO2	CATGCTTATGTGAGTCCTCCCCTTCCCGTGTGGATTATAGGGGTGACTGATAGAG
CRZ1 KO3	GTTTCTACATCTCTTCCGTGTTAATACATCGCCCGATGGTCATAGGGCGCTG
CRZ1 KO4	GGAGATTGCCTTTCCCGACTTTGACTTG
CRZ1 KO5	GTATTCGGGCGCGCGAATTTGT
CRZ1 KO6	GGGATCGGGGCGGGGTATGAGGC
VPS45 probe F	CGAAAGCCAAGATGGATGAG
VPS45 Probe R	CGACAGTGGTGATGAAGAAC
Vps45-XHO1 F	TGCTCTCGAGTTTGTTCATAAAATATCAGGCGGCTTT
VPS45-XHOI R	GATTCTCGAGTAATCAGCATGCAAGATCCAGCATTGATAC
VPS45 end + Linker 2R	AGAACATTGACCCTGAGGAGTGCCTCCTCTACCAGAACCACCAG

Safe Haven Fusion 2F	GAGGGTTAATTGCGCGCTTGGCG
pHD58_GFP_linker_F	TCTGGTGGTTCTGGTTCTGTGAGCAAGGGCGAGGAGCT
Safe Haven 2R	CGCCAAGCGCGCAATTAACCCTC
