# Supplementary figures and images for: The Sec1/Munc18 (SM) protein Vps45 is involved in iron uptake, mitochondrial function and virulence in the pathogenic fungus Cryptococcus neoformans
Source: PLoS Pathog. 2018 Aug 2;14(8):e1007220. doi: 10.1371/journal.ppat.1007220 (PMC6091972; doi:10.1371/journal.ppat.1007220)

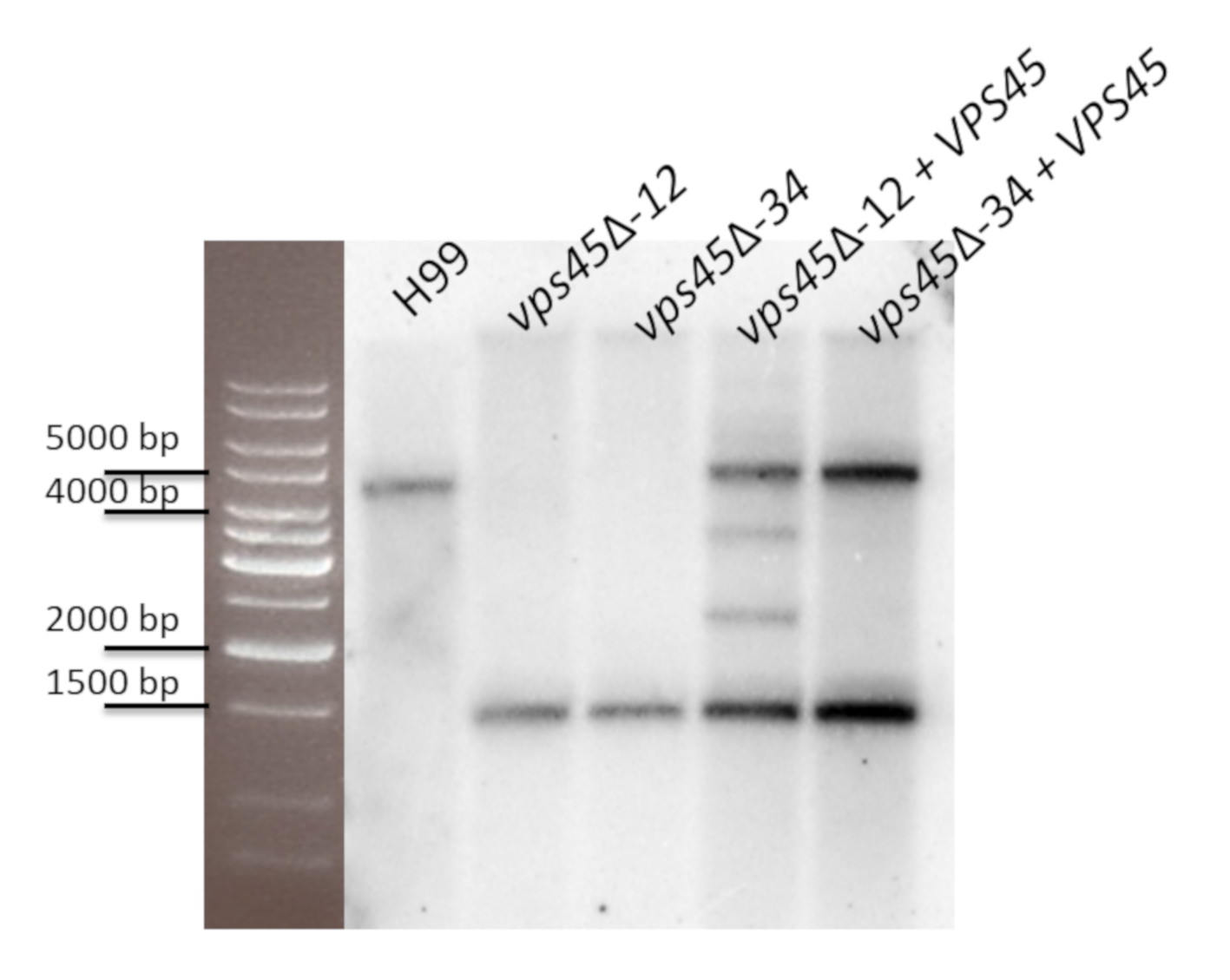

Supplement: S1 Fig — Extracted DNA was digested with BglI and genomic hybridization to detect the VPS45 locus yielded fragments of 4329 bp in the WT strain, 1582 bp in the deletion mutants, and 4478 bp and 1582 bp in complemented strains. (TIF) [file ppat.1007220.s002.tif]

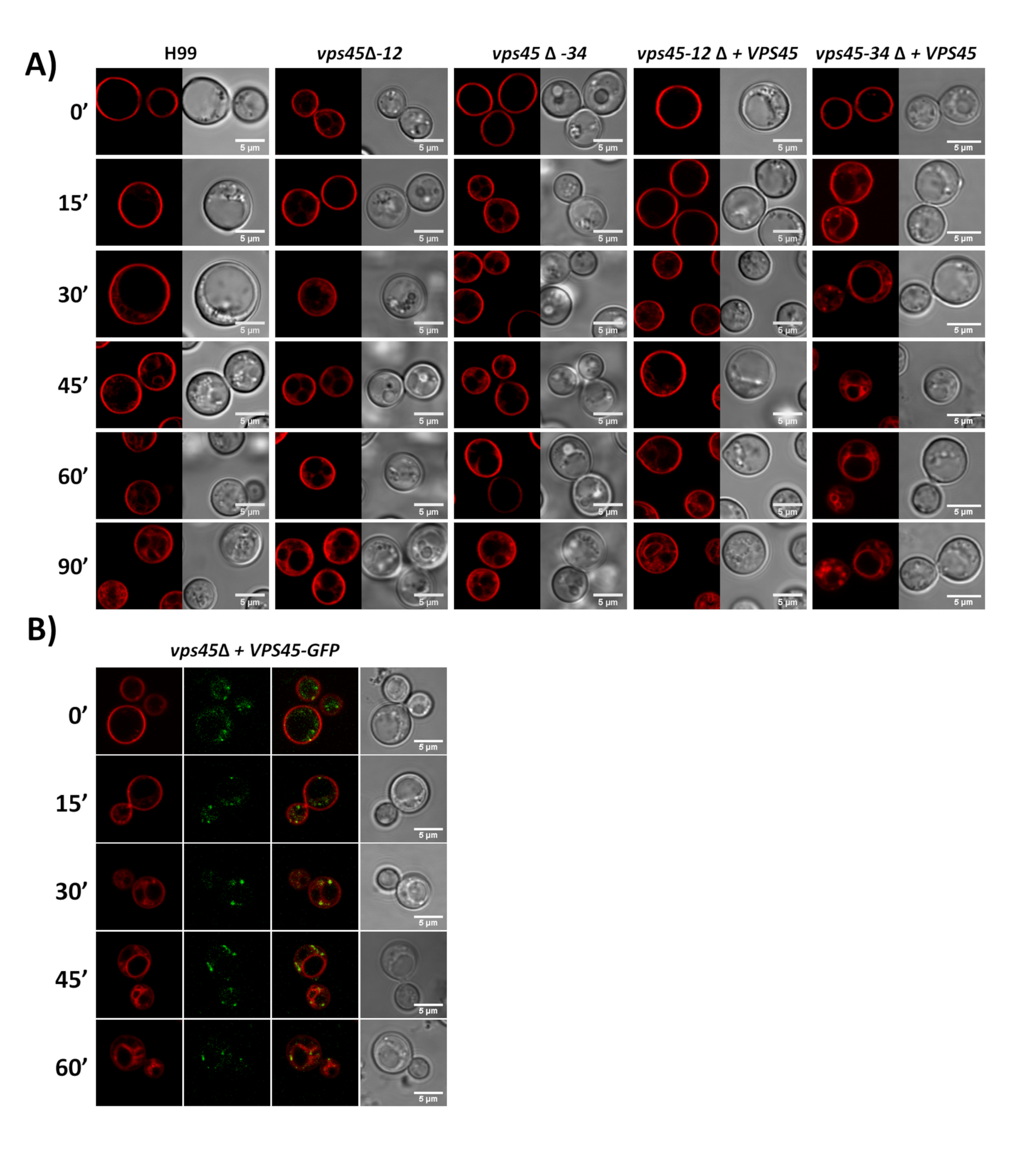

Supplement: S2 Fig — WT, vps45 mutant, complemented (A), and GFP-tagged (B) strains were grown for 24h in YNB-BPS at 37°C. 1X106 cells/mL were inoculated in YNB-BPS + 100μM FeCl3, stained with 5 μM FM4-64 and transferred in a chamber slide where the cells were maintained at 37°C. Confocal microscopy images were taken every 15 min for 90 minutes. Colocalization analyses using ImageJ coloc2 test revealed a positive correlation between VPS45-GFP and endocytic membranes as determined by Pearson’s R value (0.15–0.45) and Costes P-value (0.99–1.00). (TIF) [file ppat.1007220.s003.tif]

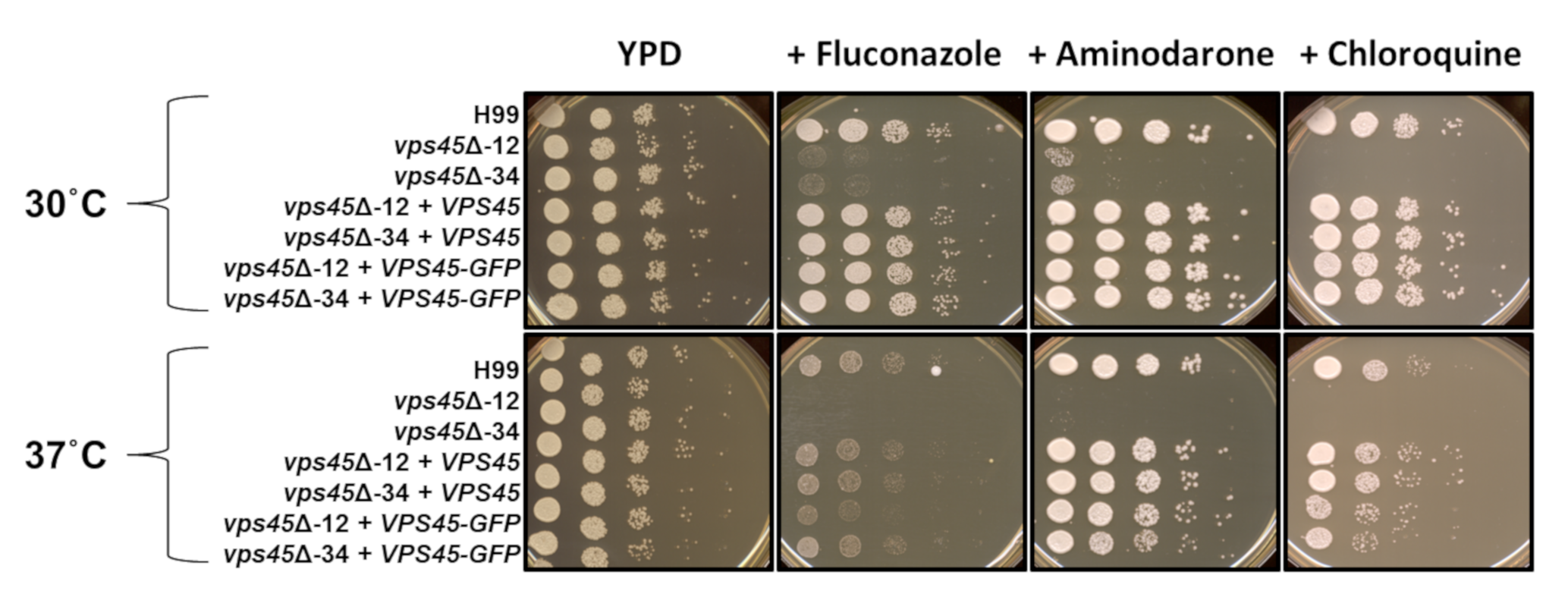

Supplement: S3 Fig — The WT, mutants and complemented strains (VPS45 or VPS45-GFP) were grown on YPD in the presence of 10μg mL-1 fluconazole, 25μM aminodarone and 6mM chloroquine. Cells were pre-cultured in YPD overnight at 30°C, serial diluted, and 5μL were spotted onto YPD plates containing the indicated drugs. Plates were incubated at 30°C or 37°C for 2 days. (TIF) [file ppat.1007220.s004.tif]

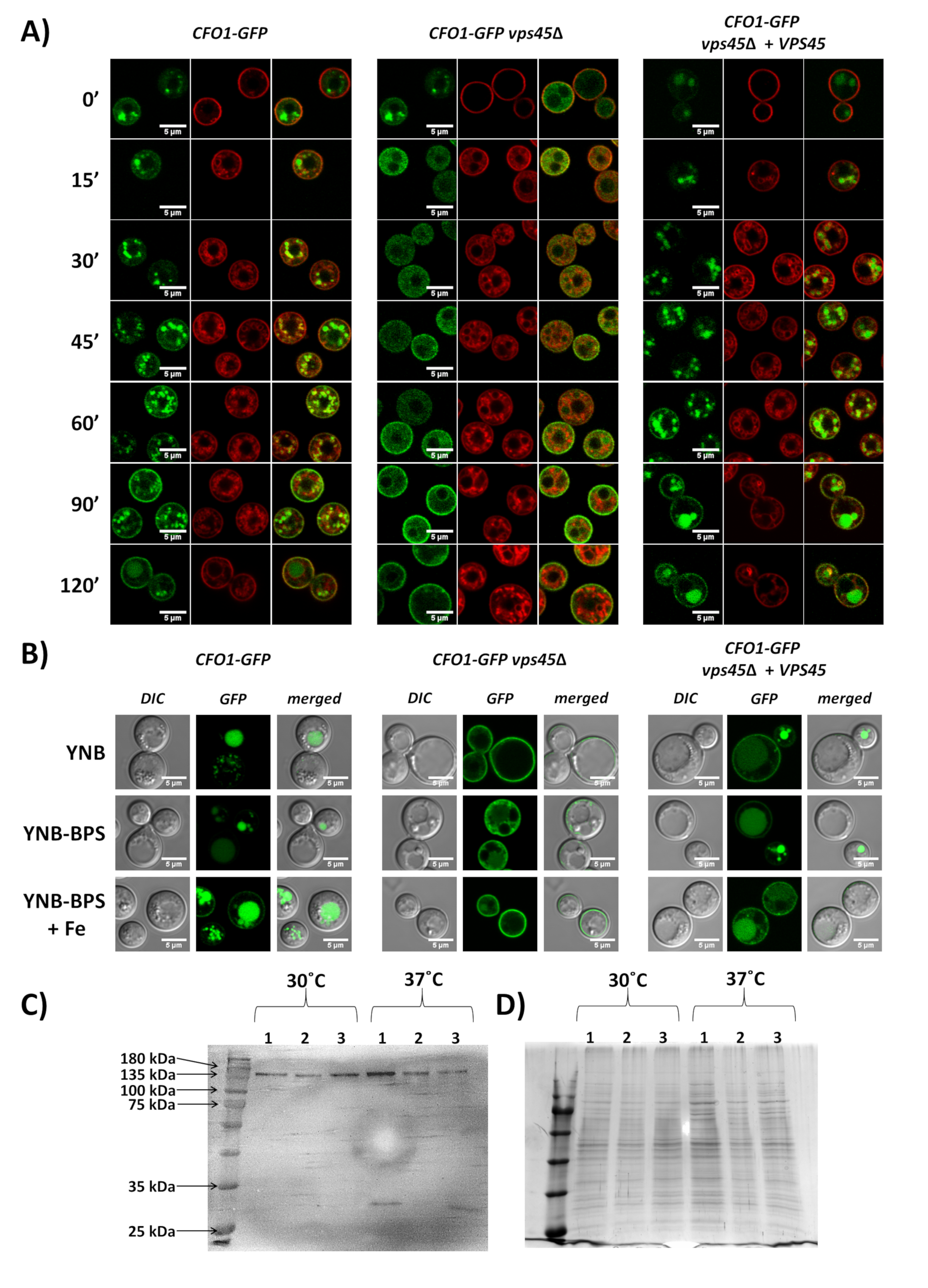

Supplement: S4 Fig — A) Localization of Cfo1-GFP in the absence of VPS45 at 37°C. Strains (WT, vps45Δ and complement) containing a CFO1-GFP construct were cultured overnight in YPD, washed 3 times and counted. 1X106 cells/mL were inoculated in YNB-BPS and incubated at 37°C for 1h. Then cells were stained with 5 μM FM4-64, transferred in a chamber slide and maintained at 37°C. Confocal images were taken every 15 minutes for 2h. B) Localization of Cfo1-GFP after 24h incubation in YNB, YNB + 150μM BPS, and YNB-BPS + 100μM FeCl3 at 37°C. The GFP label indicates evaluation of the green fluorescent protein and DIC indicates differential interference contrast microscopy. C) Visualization of full-length Cfo1-GFP by Western Blot in the WT strain (1), the vps45 mutant (2) and the complemented strain (3) cells incubated for 3h in YNB+150μM BPS at 30°C or 37°C. Western Blot on Cfo1-GFP using the mouse monoclonal GFP antibody (B-2) (Santa Cruz Biotechnology). Cfo1 is expected to be glycosylated like its homologue Fet3p in S. cerevisiae and this would explain the observed slower migration of the band (~140 kDa) versus the predicted size of the fusion (~100 kDa; Cfo1: 71.1 kDa and GFP: 27 kDa) (59). D) Blue silver staining of the SDS-PAGE gel with ~10 ug of total cell protein extracted by bead beating and sonication from cells incubated for 3h in YNB + 150μM BPS at 30°C or 37°C. (TIF) [file ppat.1007220.s005.tif]

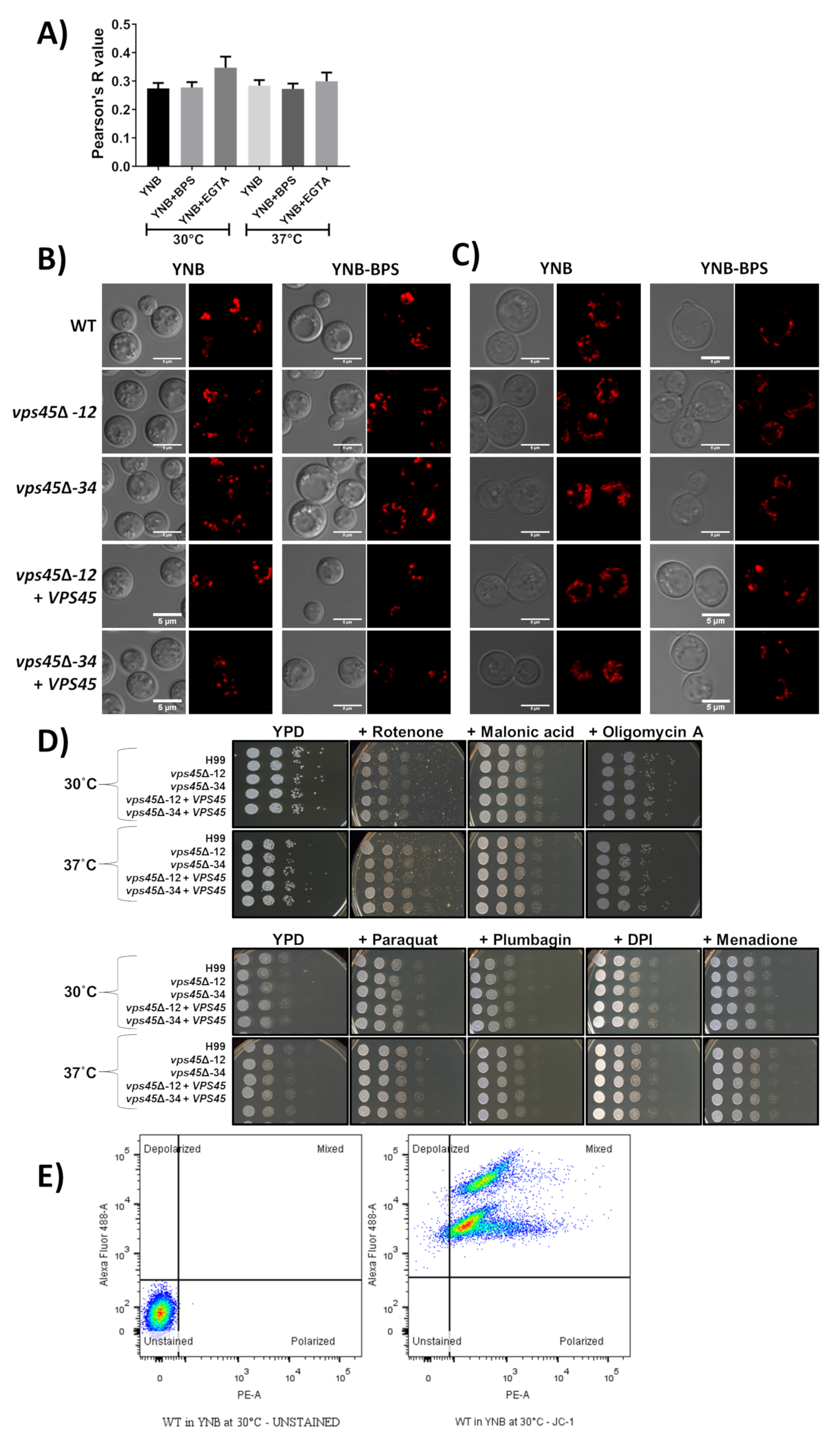

Supplement: S5 Fig — A) Mean R values of positive correlation between VPS45-GFP and mitochondria of cells grown under different conditions. Colocalization analyses were performed on images of whole cells (15–45 cells) using the ImageJ coloc2 test. Positive correlations were determined by Costes P-value (0.95–1.00). Disruption of VPS45 did not influence mitochondria morphology in iron-chelated conditions. Confocal images of mitochondria stained with 500nM mitotracker were taken of the WT, mutant and complemented strains inoculated for 24h in YNB ± 150μM BPS at 30°C (B) and 37°C (C). D). The WT, mutants and complemented strains were grown in the presence of inhibitors of the mitochondrial electron transport chain and ROS. Cells were pre-cultured in YPD overnight at 30°C, serial diluted, and 5μL were spotted onto YPD plates containing 75μg mL-1 rotenone, 1mM malonic acid, 2μg mL-1 oligomycin A, 500μM paraquat, 50μM plumbagin, 50μM diphenyleneiodonium chloride (dpi) or 5μg mL-1 menadione. Plates were incubated at 30°C and 37°C for 2 days. E) Example of flow cytometry assessment of mitochondrial membrane potential with the JC-1 dye. The percentages of cells with polarized, mixed and depolarized mitochondria membrane potential were determined by gating the fluorescence of unstained single cells population to stained single cells population. (TIF) [file ppat.1007220.s006.tif]

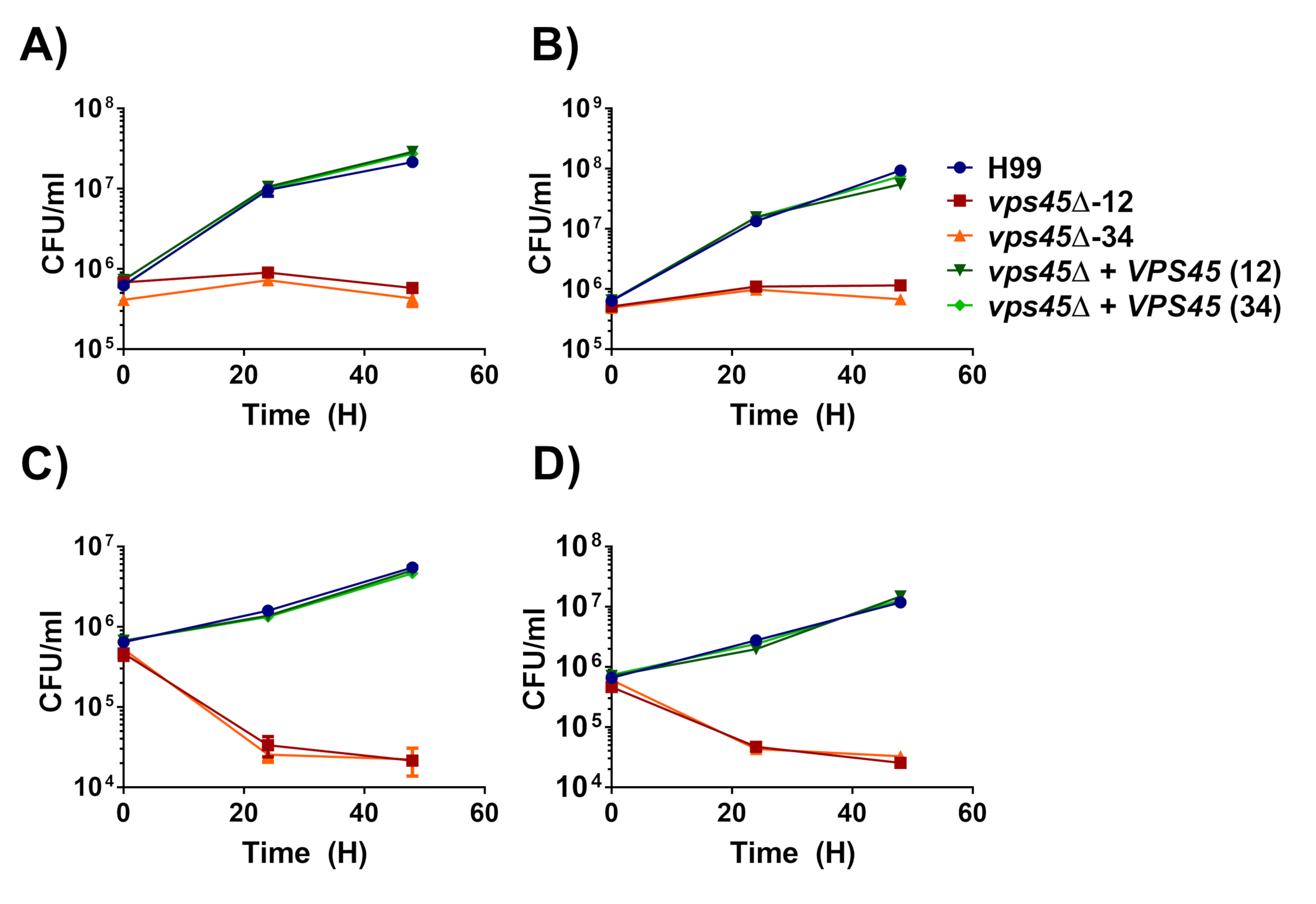

Supplement: S6 Fig — Cells were pre-grown in defined LIM for 48h at 30°C, washed and counted. 5X105 cells/mL were inoculated into LIM (A and C), LIM + 100 μM FeCl3 (B and D) and incubated at 30°C (A and B) or 37°C (C and D) for 48h. (TIF) [file ppat.1007220.s007.tif]
